# The emergence of *Klebsiella pneumoniae* liver abscess in non-diabetic patients and the distribution of capsular types

**DOI:** 10.1186/s13099-016-0128-y

**Published:** 2016-10-18

**Authors:** Chien Chuang, Wen-Chien Fan, Yi-Tsung Lin, Fu-Der Wang

**Affiliations:** 1 Department of Medicine, Taipei Veterans General Hospital, Taipei, Taiwan; 2 Division of Infectious Diseases, Department of Medicine, Taipei Veterans General Hospital, Number 201, Section 2, Shih-Pai Road, Beitou District, Taipei, 11217 Taiwan; 3 School of Medicine, National Yang-Ming University, Taipei, Taiwan; 4 Institute of Emergency and Critical Care Medicine, National Yang-Ming University, Taipei, Taiwan

**Keywords:** Capsular type, Diabetes, Hemoglobin A1c, *Klebsiella pneumoniae*, Liver abscess

## Abstract

**Background:**

*Klebsiella pneumoniae* is the most common pathogen of community-acquired pyogenic liver abscess in East Asia. Diabetes mellitus (DM) is a well-established risk factor for *K. pneumoniae* liver abscess (KPLA). However, reports regarding the emergence of KPLA in non-diabetic patients are limited.

**Results:**

A total 230 patients with KPLA from a medical center in Taiwan were identified retrospectively. The rate of DM in patients with KPLA was 44.4 % in 2011, 57.9 % in 2012, 44.9 % in 2013, 35.0 % in 2014, and 53.5 % in 2015. Diabetic patients had higher rate of gas-forming abscesses than non-diabetic patients, but the clinical outcomes were not different. The six virulent capsular types (K1, K2, K5, K20, K54, and K57) accounted for 90.2 % of all *K. pneumoniae* isolates, and were more prevalent in non-diabetic than diabetic patients (93.9 vs 85.9 %, *P* = 0.048). The six virulent capsular types were also more prevalent in the group with optimal glycemic levels (Non-DM and DM with HbA1c level <7 %) than the DM group with HbA1c level ≥7 % (93.9 vs 84.3 %, *P* = 0.022).

**Conclusion:**

*Klebsiella pneumoniae* liver abscess has emerged in non-diabetic patients in Taiwan. Diabetic patients were at higher risk of acquiring gas-forming abscesses. Non diabetic patients and diabetic patients with optimal glycemic levels are more susceptible to the virulent capsular types of *K. pneumoniae*.

## Background

*Klebsiella pneumoniae* is known as a major pathogen to cause nosocomial urinary-tract infection, pneumonia, and intra-abdominal infections worldwide [1–3], as well as community-acquired pyogenic infection in Taiwan [4–6]. In the past three decades in East Asian countries (especially Taiwan, Korea, and Singapore), *K. pneumoniae* has emerged as the major cause of community-acquired pyogenic liver abscess [7–15]. A distinct invasive syndrome of *Klebsiella pneumoniae* liver abscess (KPLA) with extrahepatic complications involving endophthalmitis, infection in the central nervous system as well as septic metastatic lesions in other organs, have also been reported [16–18]. The prevalent KPLA is now considered as an endemic disease in Taiwan [14, 19]. The mortality rate due to KPLA has decreased in Taiwan in recent years [14, 16]. However, metastatic complications with ocular or neurological involvement from KPLA could result in catastrophic disability and a poor long-term prognosis [18]. The high economic burden resulting from KPLA is also the concern [9].

Diabetes mellitus (DM) is a well-established risk factor for KPLA. The percentage of DM in KPLA patients was as high as 60–78.4 % in previous studies in Taiwan [12, 16, 17, 20]. Patients with underlying DM are also more susceptible to septic metastatic complications from KPLA [12, 17, 20, 21]. Recently, we reported that diabetic patients with uncontrolled glycemia were more susceptible to metastatic complications from KPLA than those with controlled glycemia [11]. In contrast to the previous studies, we found that about half of the patients did not have DM from January 2007 to January 2012 [11]. However, the emergence of KPLA in non-diabetic patients has received little attention in the literature. The relationship between the capsular types of *K. pneumoniae* and different glycemic status of patients with KPLA remains undetermined.

In this study, we aimed to investigate the clinical characteristics of KPLA patients and the associated capsular types of *K. pneumoniae* strains in Taiwan in the recent 5 years. Particular attention was focused on the emergence of KPLA in non-diabetic patients.

## Methods

### Study population and data collection

This retrospective analysis enrolled the patients diagnosed with KPLA who were admitted to Taipei Veterans General Hospital (a 2900-bed tertiary medical center) from January 2011 to December 2015. We excluded patients aged <20 years, patients with polymicrobial or nosocomial infections, or patients who developed liver abscess after invasive procedure, including trans-hepatic arterial chemoembolization or stent replacement over common bile duct. The medical records of patients with KPLA were reviewed by two infection specialists. Information regarding clinical features, underlying diseases, laboratory findings, origin and nature of the liver abscesses, imaging findings, treatment and outcomes were collected. Assessment of blood glycemic status was based on hemoglobin A1c (HbA1c) levels at the time of infection or as close to the time of infection as possible, within 1 month before this episode of KPLA. Since newly diagnosed DM is not unusual in patients with KPLA, it is a common practice to check HbA1c in patients with KPLA in Taiwan. This study was approved by Institutional Review Board of Taipei Veterans General Hospital.

### Definition

An episode of KPLA was defined as a culture-confirmed *K. pneumoniae* isolated from an abscess or blood and ≥1 liver abscess, detected by sonography or computed tomography. Only the first episode of KPLA in an individual patient diagnosed at our hospital during the study period was included. The diagnosis of DM was according to the criteria published by the American Diabetes Association in 2012: HbA1c level ≥6.5 %, symptoms of hyperglycemia with a random plasma glucose ≥200 mg/dL, or 2-h plasma glucose ≥200 mg/dL during an oral glucose tolerance test, or fasting plasma glucose ≥126 mg/dL [23]. “Cryptogenic KPLA” was that in which no obvious extra-hepatic source of infection could be identified. “Biliary-tract origin of a liver abscess” was defined if the clinical features of cholecystitis/cholangitis or extra-hepatic biliary ductal abnormalities were identified upon radiography [11]. “Chronic lung disease” included chronic obstructive pulmonary disease, bronchiectasis, or any structural lung disease with the exception of bronchogenic carcinoma. Chronic kidney disease was defined as baseline serum creatinine ≥2 mg/dL. “Metastatic infection” was defined as a distant site of infection isolated with the same pathogen (*K. pneumoniae*) as in a pyogenic liver abscess. “Multiple liver abscesses” were defined as ≥3 abscesses detected by imaging. The Acute Physiology and Chronic Health Evaluation II (APACHE II) score was estimated within 48 h after hospital admission.

### Microbiology laboratory procedures

The VITEK 2 system (bioMérieux, Marcy l’Etoile, France) or matrix-assisted laser desorption-ionization time-of-flight mass spectrometry (bioMérieux SA, Marcy l’Etoile, France) was used to confirm bacterial identifications among the available isolates. To determine the capsular types of *K. pneumoniae*, we undertook *cps* genotyping by the polymerase chain reaction (PCR) detection of K serotype-specific alleles at *wzy* and *wzx* loci, including K1, K2, K5, K20, K54, and K57, as described previously [16]. These capsular types are thought to be the most closely associated with community invasive disease or pathogenicity [24].

### Statistical analyses

Data were analyzed using SPSS ver. 17 (SPSS, Chicago, IL, USA). Chi square or Fisher’s exact tests were carried out for categorical data. The two-tailed Student’s *t* test or Mann–Whitney *U* test was done for numerical data. A *P* value < 0.05 was considered statistically significant.

## Results

### Clinical characteristics of KPLA in patients with or without DM

A total of 255 patients diagnosed with KPLA during the study period were enrolled. 25 patients were excluded: 6 patients were infected by multidrug-resistant *K. pneumoniae* nosocomially, 6 patients developed a liver abscess after trans-hepatic arterial chemoembolization for hepatocellular carcinoma, 1 patient developed a liver abscess after replacing a stent over common bile duct, and 12 patients had polymicrobial liver abscess. Of the remaining 230 patients (149 males), the mean age was 63.8 ± 15.5 years. The overall in-hospital mortality of KPLA was 3.5 % (8 patients). More than half the patients (n = 120, 52.2 %) in this study period did not have DM. The rate of DM in patients with KPLA was 44.4 % in 2011, 57.9 % in 2012, 44.9 % in 2013, as low as 35.0 % in 2014, and 53.5 % in 2015.

To further characterize non-diabetic patients with KPLA, Table 1 details the comparisons between diabetic and non-diabetic patients with KPLA. More male patients were in diabetic than non-diabetic group (72.7 vs 57.5 %, *P* = 0.016). Diabetic patients had a significantly higher Charlson comorbidity index (2.4 ± 1.9 vs 1.1 ± 1.8, *P* < 0.001) than those without diabetes. The prevalence of gas-forming abscess (16.4 vs 1.7 %, *P* = < 0.001) was significantly higher in diabetic than non-diabetic patients. The APACHE II score was significantly higher in diabetic patients (14.5 ± 7.4 vs 12.3 ± 6.9, *P* = 0.018), whereas rate of ICU admission, length of hospital stay, and in-hospital mortality did not differ between the two groups. Interestingly, capsular types K1 and K2 tended to be more prevalent in non-diabetic than diabetic patients (81.7 vs 70.7 %, *P* = 0.057).

**Table 1:** Clinical characteristics of diabetic and non-diabetic patients with KPLA

	Non-DM (n = 120)	DM (n = 110)	*p*
Age in years	64.1 ± 16.3	63.5 ± 14.6	0.779
Male	69 (57.5)	80 (72.7)	0.016
Charlson score	1.1 ± 1.8	2.4 ± 1.9	<0.001
Underlying disease			
Malignancy	11 (9.2)	10 (9.1)	0.984
Alcoholism	4 (3.3)	8 (7.3)	0.180
Chronic kidney disease	4 (3.3)	8 (7.3)	0.180
Liver cirrhosis	5 (4.2)	1 (0.9)	0.215
Congestive heart failure	8 (6.7)	5 (4.5)	0.487
Chronic lung disease	5 (4.2)	5 (4.5)	1.000
Cerebrovascular accident	11 (9.2)	8 (7.3)	0.602
APACHE II score	12.3 ± 6.9	14.5 ± 7.4	0.018
HbA1c	5.6 ± 1.2	9.2 ± 2.6	<0.001
Capsular type K1 and K2	94 (81.7)^a^	70 (70.7)^b^	0.057
Wild-type antibiotic susceptibility	113 (94.2)	103 (93.6)	0.867
Origin			0.749
Cryptogenic	103 (85.8)	96 (87.3)	
Biliary tract origin	17 (14.2)	14 (12.7)	
Abscess location			0.408
Right lobe	77 (64.2)	79 (71.8)	
Left lobe	21 (17.5)	17 (15.5)	
Both lobes	22 (18.3)	14 (12.7)	
Abscess size			0.419
<5 cm	51 (42.5)	41 (37.3)	
≥5 cm	69 (57.5)	69 (62.7)	
Gas-forming abscess	2 (1.7)	18 (16.4)	<0.001
Multiple abscesses	18 (15.0)	15 (13.6)	0.768
Metastatic infection	6 (5.0)	12 (10.9)	0.096
Outcomes			
ICU admission	23 (19.2)	28 (25.5)	0.252
Hospital days	26.9 ± 16.5	32.3 ± 38.5	0.178
Mortality	4 (3.4)	4 (3.6)	0.910

Data are presented as mean ± SD or frequency with percentage (%)

^a^115 isolates were available for genotyping

^b^99 isolates were available for genotyping

### Clinical characteristics of KPLA in non-diabetic patients and diabetic patients with optimal glycemic level

We compared the clinical characteristics between non-diabetic patients and diabetic patients with optimal glycemic level (HbA1c <7 %) (Table 2). Diabetic patients with optimal glycemic level had significantly higher Charlson comorbidity index than those without diabetes (2.0 ± 1.4 vs 1.1 ± 1.8, P = 0.047). Interestingly, gas-forming abscesses (15.8 vs 1.7 %, *P* = 0.018) occurred more frequently even in diabetic patients with optimal glycemic level than those without diabetes.

**Table 2:** Clinical characteristics of patients with KPLA stratified by non-diabetic and diabetic with optimal glycemic control

	Non-DM (n = 120)	DM with HbA1c < 7 % (n = 19)	*p*
Age in years	64.1 ± 16.3	69.5 ± 12.9	0.166
Male	69 (57.5)	13 (68.4)	0.369
Charlson score	1.1 ± 1.8	2.0 ± 1.4	0.047
Underlying disease			
Malignancy	11 (9.2)	3 (15.8)	0.408
Alcoholism	4 (3.3)	0 (0)	1.000
Chronic kidney disease	4 (3.3)	3 (15.8)	0.054
Liver cirrhosis	5 (4.2)	0 (0)	1.000
Congestive heart failure	8 (6.7)	2 (10.5)	0.627
Chronic lung disease	5 (4.2)	1 (5.3)	0.593
Cerebrovascular accident	11 (9.2)	0 (0)	0.361
APACHE II score	12.3 ± 6.9	14.9 ± 8.4	0.136
Capsular type K1 and K2	94 (81.7)^a^	12 (75.0)^b^	0.506
Wild-type antibiotic susceptibility	113 (94.2)	17 (89.5)	0.355
Origin			1.000
Cryptogenic	103 (85.8)	17 (89.5)	
Biliary tract origin	17 (14.2)	2 (10.5)	
Abscess location			0.937
Right lobe	77 (64.2)	12 (63.2)	
Left lobe	21 (17.5)	4 (21.1)	
Both lobes	22 (18.3)	3 (15.8)	
Abscess size			0.369
<5 cm	51 (42.5)	6 (31.6)	
≥5 cm	69 (57.5)	13 (68.4)	
Gas-forming abscess	2 (1.7)	3 (15.8)	0.018
Multiple abscesses	18 (15.0)	3 (15.8)	1.000
Metastatic infection	6 (5.0)	1 (5.3)	1.000
Outcomes			
ICU admission	23 (19.2)	3 (15.8)	1.000
Hospital days	26.9 ± 16.5	28.1 ± 21.1	0.786
Mortality	4 (3.4)	0 (0)	1.000

Data are presented as mean ± SD or frequency with percentage (%)

^a^115 isolates were available for genotyping

^b^16 isolates were available for genotyping

### Distribution of *K. pneumoniae* capsular genotypes in patients with different glycemic levels

Among 230 patients with KPLA, 214 isolates were available for capsular genotyping. Table 3 shows the distribution of capsular types in patients with different glycemic levels. Overall, the six virulent capsular types (K1, K2, K5, K20, K54, and K57) accounted for 90.2 % (n = 193) of all *K. pneumoniae* isolates. Capsular type K1 (n = 121, 56.5 %) and K2 (n = 43, 20.1 %) were the most prevalent strains. We also found that 54.5 % (66/121) K1 isolates and 65.1 % (28/43) K2 isolates were isolated from non-diabetic patients, respectively. Capsular type K1 and K2 isolates had the trend to be more prevalent in non-diabetic than diabetic patients (81.7 vs 70.7 %, *P* = 0.057). The six virulent capsular types (K1, K2, K5, K20, K54, and K57) were significantly more prevalent in non-diabetic than diabetic patients (93.9 vs 85.9 %, *P* = 0.048).

**Table 3:** Distribution of capsular types of *K. pneumoniae* isolates in non-diabetic and diabetic patients with different HbA1c levels

Genotype	Non-DM (n = 115)	DM		*p* ^a^	*p* ^b^
		HbA1c **<** 7 % (n = 16)	HbA1c ≥ 7 % (n = 83)		
K1	66 (57.4)	12 (75.0)	43 (51.8)	0.787	0.266
K2	28 (24.3)	0 (0)	15 (18.1)	0.094	0.557
K5	6 (5.2)	0 (0)	3 (3.6)	0.510	1.000
K20	1 (0.9)	1 (6.3)	4 (4.8)	0.098	0.210
K54	4 (3.5)	1 (6.3)	4 (4.8)	0.736	0.737
K57	3 (2.6)	1 (6.3)	1 (1.2)	1.000	0.651
The above 6 capsular types	108 (93.9)	15 (93.8)	70 (84.3)	0.048	0.022
Others	7 (6.1)	1 (6.3)	13 (15.7)	0.048	0.022

Data are presented as frequency with percentage (%)

^a^Comparison between Non-DM and DM

^b^Comparison between group with optimal glycemic level (Non-DM + DM with HbA1c **<** 7 %) and diabetic patients without optimal glycemic level (DM with HbA1c level ≥ 7 %)

To further explore the association of glycemic status with the distribution of capsular types, non-diabetic and diabetic patients with HbA1c level <7 % were defined as the group with optimal glycemic level. Diabetic patients with HbA1c level ≥7 % were defined as the non-optimal glycemic level group. We found that the six virulent capsular types (K1, K2, K5, K20, K54, and K57) were significantly more prevalent in the group with optimal glycemic level than the group without optimal glycemic level (93.9 vs 84.3 %, *P* = 0.022).

## Discussion

The present study demonstrated the emergence of KPLA in non-diabetic patients as well as the distribution of capsular types of *K. pneumoniae* in Taiwan. In our 5-year cohort study, more than half of the patients did not have underlying DM. While the rate of gas-forming abscess was significantly higher in diabetic than non-diabetic patients, the clinical outcomes did not differ. Capsular types K1, K2, K5, K20, K54, and K57 accounted for the majority of all the *K. pneumoniae* isolates. These six virulent capsular types were significantly more prevalent in the group with optimal glycemic level than that with non-optimal glycemic level.

In one study conducted from July 1991 to June 1998 in two medical centers (including our hospital) in Taiwan, DM was present in 78.4 % (105/134) of patients with KPLA [12]. In one recent review article, 63 % (323/512) of patients with KPLA had underlying DM in Taiwan [22]. However, in our present study, DM accounted for only 47.8 % (110/230) of KPLA patients in the previous 5-year period (2011–2015). These findings further imply that DM is not a prerequisite for the development of KPLA anymore. As an endemic disease in Taiwan, physicians should be vigilant about the potential of acquiring KPLA in patients without underlying DM. Despite the emergence of KPLA in non-diabetic patients, we still found a higher rate of gas-forming abscess in diabetic patients. This finding corresponded to our former study that indicated the important role of glycemic level in clinical characteristics of KPLA [11].

The comparison of clinical characteristics between non-diabetic patients and diabetic patients with optimal glycemic level (HbA1c <7 %) has never been addressed. We found that gas-forming abscess was significantly more prevalent in diabetic patients with optimal glycemic levels than those without diabetes. But the clinical outcome were not different between these two groups. These results might suggest that previous glycemic exposure still had adverse effects upon immunity against subsequent bacterial infection.

The literature has demonstrated that K1 and K2 were the major capsular types causing KPLA [22]. Fang et al. has demonstrated that six capsular types (K1, K2, K5, K20, K54, and K57) accounted for 92 % of the *K. pneumoniae* isolates during 1997–2005 in a medical center in Taiwan [16]. Similarly, our study disclosed that the six capsular types accounted for 93.9 % of *K. pneumoniae* isolates. However, reports regarding the impact of glycemic status on the distribution of capsular types of *K. pneumoniae* isolates are limited. In the current study, K1 isolates predominated in non-DM group and DM groups with different HbA1c levels. This result again documented that capsular type K1 is the major virulent factor causing KPLA. Notably, we found that 65.1 % (28/43) of all K2 isolates occurred in non-diabetic patients, which was higher than the previous report (4/10, 40 %) by Yu et al. [25]. These findings imply that *K. pneumoniae* capsular type K2 is sufficiently virulent to cause severely invasive infection, even in a healthy host. Whether or not the prevalent K2 strains account for the increasing proportion of KPLA in non-diabetic patients remained uncertain. Further studies to explore this issue are needed.

We also found that six virulent capsular types were more prevalent in non-DM than DM group. In terms of the association of glycemic status with the distribution of capsular types, the group with optimal glycemic level (Non-DM and DM with HbA1c level <7 %) was infected with these six virulent capsular types more frequently than the DM group with HbA1c level ≥7 %. It implies that non-DM or healthy persons with KPLA are infected primarily with virulent *K. pneumoniae* capsular types, whereas other capsular types with a relatively lower virulence can still cause liver abscesses, mostly in DM patients without optimal glycemic control [25].

Our study has some limitations. First, this study was conducted in a tertiary medical center in northern Taiwan, and all data were collected from medical records retrospectively. Further studies should be carried out prospectively in other regions or countries to confirm our findings. Second, not all *K. pneumoniae* isolates were determined for capsular genotyping. Finally, the number of diabetic patients with optimal glycemic level was relatively small in comparison to other groups. Despite these limitations, our study was the first to identify the distribution of *K. pneumoniae* capsular types in patients with KPLA according to the different glycemic status.

## Conclusions

In conclusion, DM accounted for less than 50 % of KPLA patients in the 5-year cohort in Taiwan. Gas-forming abscesses from KPLA occurred more frequently in diabetic patients. The prevalent virulent capsular types of *K. pneumoniae* in patients with optimal glycemic level play an important role in the emergence of KPLA in non-diabetic patients. Vaccination for prevention of KPLA is not available currently in Taiwan, and it is expected that the virulent *K. pneumoniae* isolates will continuously occur in hosts without traditional risk factors. Further study with emphasis on the characteristics of KPLA in non-diabetic patients is warranted. The knowledge of distribution of capsular types among patients with different glycemic levels will be crucial for the disease control and prevention in the future.

## Abbreviations

DM: diabetes mellitus
KPLA: *Klebsiella pneumoniae* liver abscess
HbA1c: hemoglobin A1c
APACHE II: Acute Physiology and Chronic Health Evaluation II
